# Cincinnati and St. Louis

**Published:** 1848-01

**Authors:** 


### CINCINNATI AND ST. LOUIS.

We think, with our friend and colleague of St. Louis, that a flourishing city, with a population of over 60,000, should certainly have at least one good Cutler. Placing the Queen City at tre le that amount—which perhaps overrates a little our present enumeration—and we have one to every 60,000 inhabitants. It is remarkable, as Dr. Brown observes, that St. Louis should be without any, for in extent of country to furnish and number of Physicians and Dentists to supply, we should think that two or three good workmen would be able to find constant employ. It is with much pleasure that we can refer our brethren of the west to our excellent cutlers of this city. We know of none who we think can excel, them in the manufacture of many surgical, as well as dental instruments.

We are glad to know that their skill is so well appreciated that their constant labor cannot supply the demand. Hence we have our Dental Depot, where every thing in the Dental line is to be had. And in connection with this subject, we would remark that our friend Mr. Leslie maintains a high reputation for the manufacture of gold foil. Indeed, scarce any thing which the Dentist may require, which can be had in the east, but can be as well supplied in Cincinnati. The trade in this line is extending very rapidly, and we hope will soon embrace the entire west.—Cin. Ed.

